# A Critique of the Undergraduate Nursing Preceptorship Model

**DOI:** 10.1155/2012/248356

**Published:** 2012-05-08

**Authors:** Monique Sedgwick, Suzanne Harris

**Affiliations:** Faculty of Health Sciences, University of Lethbridge, Lethbridge, AB, Canada T1K 3M4

## Abstract

The preceptorship model is a cornerstone of clinical undergraduate nursing education in Canadian nursing programs. Their extensive use means that nursing programs depend heavily on the availability and willingness of Registered Nurses to take on the preceptor role. However, both the health service and education industries are faced with challenges that seem to undermine the effectiveness of the preceptorship clinical model. Indeed, the unstable nature of the clinical setting as a learning environment in conjunction with faculty shortages and inadequate preparation for preceptors and supervising faculty calls us to question if the preceptorship model is able to meet student learning needs and program outcomes. In a critical analysis of preceptorship, we offer a deconstruction of the model to advance clinical nursing education discourse.

## 1. Introduction

A review of the nursing education literature reveals that clinical nursing education is considered to be a vital component of nurses' education. Indeed, Florence Nightingale, the founder of contemporary nursing practice, placed clinical education at the center of nurses' professional development. According to Nightingale, nurses' first year of training should occur in the hospital setting under the direct supervision of practicing nurses who can guide neophytes in the care of their patients [1]. While this type of clinical education model was the prototype of what would later be called preceptorship, the model remained largely dormant during hospital-based programs until it emerged once again in the 1960s in nurse practitioner programs [1]. In Canadian undergraduate nursing programs, preceptorship is typically described as a formal one-to-one relationship between a nursing student and registered nurse that extends over a pre-determined length of time [2]. Since the 1980s it has become a cornerstone of clinical nursing education. Given that the use of the preceptorship model is extensive, most Canadian programs are somewhat to very dependent on preceptors to guide their students [3]. Furthermore, because of its wide use, many nurse scholars believe that preceptorship provides the perfect medium to bridge theory and practice [1] and a way to facilitate the transition from student to graduate nurse role for the majority of nursing students [4].

 However, from an education and health sector perspective there is still significant concern about the clinical learning and teaching components of undergraduate nurse education [5]. Indeed, ongoing restructuring within the Canadian health care system juxtaposed with mandated increased seats in nursing programs has taxed clinical practice settings beyond their capacity. The unstable nature of the clinical practice setting as a learning environment coupled with the challenges associated with a faculty workforce shortage illuminates the limitations of the preceptorship model of clinical instruction. These limitations ultimately create challenges for students being able to meet program objectives. Given that there continues to be a lack of criteria for determining what constitutes effective clinical education [6, 7], the purpose of this paper is to generate discussion by providing a critique of the preceptorship model of clinical undergraduate nursing education.

## 2. Background

 In Canada and elsewhere, the need for nurses to be prepared at the baccalaureate level is well established and no longer disputed [8, 9]. The shift from hospital-based nursing education programs to university and/or college educational settings has resulted in a giving way of educational models grounded in behaviourism to those based on interpretative pedagogies (feminism, phenomenology, postmodernism, and critical inquiry) and humanism [7, 10]. A shift in clinical nursing education has also ensued since faculty-supervised practicum is largely unsustainable [11]. Innovative approaches to clinical nursing education have been introduced into nursing programs resulting in at least ten different models of clinical education including preceptorship currently being used in entry-level nursing education programs [12]. In their overview of these clinical education models, Budgen and Gamroth [12] note key differences among them including differing ratios of students to teachers; variations in roles and responsibilities among students, faculty and nursing staff in relation to student supervision, teaching and learning, and evaluation; differences in the nature of relationships between practice and academia; variations in the implementation of these models. While these authors conclude that all models have inherent benefits and limitations that could be maximized or minimized depending on implementation, clarity of roles and responsibilities is central.

### 2.1. Preceptorship

 While undergraduate nursing students are most often preceptored in the final semester of their program [13], sometimes they are preceptored earlier in their program [14, 15]. For this discussion, our comments will be limited to preceptorship that occurs in the final semester of Canadian nursing programs. In order to set the context for this critique, a general description of the preceptorship model in the final semester is offered.

According to the Canadian Nurses Association [2] preceptorship is intended to assist students in acquiring a basic level of knowledge, skills, and personal attributes as well as to be socialized into the profession. To facilitate being able to meet these goals as well as nursing program goals, every effort is made to have a 1 : 1 student-to-preceptor ratio. However, variations in this ratio exist. Students usually work the same hours as the preceptor with the assumption that they will progress to assuming the full nursing role and workload of the preceptor. Last, selection and preparation for the preceptor role vary across nursing programs and health regions.

 For various reasons, preceptorship has become prevalent in Canadian nursing education programs as a complimentary clinical teaching model to the traditional faculty-supervised model of instruction. Economically, preceptored clinical experiences require fewer faculty members for supervision than in traditional faculty-supervised practica. This reduces expenditures and helps to contain nursing program costs [16]. Although preceptorships maximize opportunities for development of confidence and professional socialization and knowledge and skill acquisition for students and preceptors [17], they also have the potential for enhancing academic-service partnerships [18].

 While an extensive body of literature pertaining to various aspects of preceptorship exists, few quantitative studies have been conducted that demonstrate the effectiveness of preceptored clinical experiences [16]. Further, within these quantitative studies there is a lack of consensus regarding the benefits or advantages of using a preceptorship model over the traditional faculty-supervised clinical model [19]. It is perhaps fitting then that we critically examine the preceptorship model of clinical instruction so that we can move toward an evidence-based clinical nursing education model [9].

## 3. Current Challenges for the Preceptorship Model of Clinical Instruction

 Although Canadian nursing programs are faced with many challenges, some of these challenges may very well undermine the effectiveness of the preceptorship model of clinical education. Indeed many programs are faced with organizational and operational challenges. For example, inconsistent selection practices and preparation of preceptors [19] as well as pressures to conform to the curriculum and traditional academic calendar [20] might very well mean that programs have little control over the quality of the learning experience. Furthermore, because the clinical setting is characterized by high patient acuity levels, shorter patient hospital stays, staff shortages coupled with an increased casualization of the workforce, mandatory overtime, and a heavier workload, we are left to wonder if the current healthcare setting is an optimal learning environment [21]. Not only are nursing programs faced with a limited number of clinical placements, but some researchers also report that ineffective use of students' time and varying quality of learning opportunities might be offered by clinical sites [22]. Last, nursing programs are faced with a faculty shortage that may mean that faculty members do not have the breadth of expertise required to provide the clinical teaching and supervision students need and preceptors want [23]. It is a concern then that regardless of Lusted's [24] call to critically examine teaching and learning practices and, by extension, clinical nursing education models, schools of nursing are becoming increasingly dependent on the availability of preceptors as students complete their program and more experiences are sought for them in highly specialized practice areas [3].

## 4. Deconstructing the Preceptorship Model of Clinical Instruction

 A 2004 report on nursing education in Canada [3] described models of clinical practice from a survey of 65 nursing programs. Superiority of the educational model was the most commonly occurring response for choice of the preceptorship model for clinical practice. According to Myrick and Yonge [1] there are three key players in the preceptorship model—the nursing student/preceptee, the preceptor (typically a registered nurse), and the supervising faculty member. Each member of this triad plays a critical part in the success of the preceptorship experience. While these authors also acknowledge that the clinical environment that includes everyone who interacts with students is important, we suggest the education institution and clinical environments are equally important players in the preceptorship model.

### 4.1. Education Institution Environment

 Since the clinical learning environment is the single most important resource in the development of competent, capable, and caring nurses [25], Canadian nursing programs typically devote in excess of 50% of their program funds to clinical education [7]. The nature of preceptorship where the faculty-to-student ratio is 1 : 15–20 helps to contain financial costs associated with clinical education. However, there are some “hidden” costs that impact the effectiveness of the preceptorship model of nursing clinical education. For instance, students are frequently asked and are subsequently placed in their preferred clinical setting that may be at great geographical distances from the educational facility. Hence, supervising faculty must commute to clinical sites to perform in-person learning assessments if time and program budgets allow and/or use more “nontraditional” ways to interact with students and preceptors alike.

Historically “non-traditional” methods of interacting with students and preceptors included telephone calls. In today's environment how we communicate with others is influenced by electronic technology, and faculty members might rely more heavily on electronic messaging such as email, text messaging, and social networks to communicate with students and preceptors. Although relying on computer-assisted technology is in keeping with the Canadian Association of Schools of Nursing [26] and the Canadian Nurses Association's [27] endorsement for the use of information and communication technologies (ICTs), in undergraduate nursing programs less than 1/2 of the nursing schools in Canada have supportive policies for integration of ICT in nursing education. Given that there is evidence that faculty visits frequently do not meet student and preceptor needs [23], the lack of supportive policies regarding ICT may hamper the quality and quantity of interaction between faculty, students, and preceptors.

 Last, although the need for preparation for clinical instruction is well documented [28], program expectations place considerable demands on supervising faculty and leave little time for preparation and critical reflection on the preceptorship model. While we do not presume to direct schools of nursing in determining faculty workload and teaching assignments, there are some practices that might impact the effectiveness of this model. For example, programs may lack knowledge of the clinical site and the population it serves [29]. Because programs might have little to no control over how preceptors are selected, they might also lack information about the preceptors themselves. Consequently, faculty may be unaware of the preceptors' teaching and learning needs and therefore are unable to effectively support preceptors in their teaching role. Programs might also lack information to give preceptors including how to evaluate students [30]. Further, faculty may also be responsible for arranging placements and scheduling of students' work hours [31] while concurrently needing to respond to other academic demands.

 Enmeshed within the practicalities of faculty workload and school policy that impact how faculty might operationalize their supervisory role during preceptorship, most nursing programs are faced with a faculty shortage [32, 33]. Hence, despite the importance of clinical education, educational facilities are relying more and more on sessional faculty to teach their clinical programs [34–38]. Though sessional faculty might be expert clinicians, clinical expertise is not equivalent to teaching effectiveness [35, 36]. Given that sessional instructors are often “parachuted” into their clinical teaching assignment, orientation to the preceptorship model and its outcomes and strategies to support student and preceptor teaching and learning might be minimal.

### 4.2. Supervising Faculty

 While the need for faculty involvement in the preceptorship experience has been reported in the nursing literature [23], the need to increase their involvement has also been reported [39–41]. However, the indirect supervisory nature of preceptorship where faculty are not involved in the daily teaching of the preceptored student might result in faculty role confusion [42]. If faculty are unclear as to the purpose of their clinical site visits, they may be unable to explore in depth with students and preceptors factors that influence student learning.

 Indeed, the “how” to teach within the preceptored experience is dependent on supervising faculty competency in four areas: awareness of students' knowledge, skill, preferred learning style, and expectations [30]; effective use of questioning [43, 44]; ICT competencies [45]; effective communication and feedback skills [7]. Since faculty may have no formal educational preparation related to clinical nursing education, not knowing “how” to teach students in a preceptored experience would present a substantial barrier in their ability to be effective.

### 4.3. Clinical Environment

 The primary function of the health care setting is to facilitate health and healing by meeting the preventative, curative, and palliative health needs of the population. To do this, the health sector requires an adequately prepared workforce in sufficient numbers to meet service needs. Health care consumers expect competent care from nurses and so expect that registered nurses will be properly prepared. In order to gain practical, real-life experience and to integrate knowledge and develop the clinical skills needed for successful professional practice [20], nursing students are placed within the health care setting. Hence, the health care setting serves a dual role, albeit a secondary role [46] where it also becomes an educational setting.

 Although leaders in the clinical setting acknowledge the importance of clinical education, clinical placements including preceptorship are not without problems [47]. Since healthcare settings focus on workplace goals and outcomes rather than on student learning [48], they are not always ideal learning environments even though they impact teaching and learning [1]. Indeed, as noted above the scarcity of clinical placements, higher patient acuity levels, and shorter patient hospital stays are factors that increase nursing staff stress [1, 21, 48]. While a cornerstone of the preceptorship model in the final semester of the students' program is to provide them with the opportunity to consistently work alongside one preceptor, staff shortages, increased casualization of the nursing workforce, mandatory overtime, and increased workloads have the potential to impact opportunities for students to work consecutive days with one preceptor [49]. Indeed, it is not unusual for students to be assigned multiple preceptors or to be “buddied” with other staff members during their preceptorship. While it is understandable that the presence of nursing students in the clinical setting might be perceived as a stressful burden [50], these challenges may also mean there is limited time for teaching and feedback, which could result in students lacking or missing out on learning opportunities [51] and complicate the efforts of registered nurses in supporting student learning.

### 4.4. Preceptor Role

According to Luhanga et al. [49], the one-to-one relationship between a student and preceptor is essential in assisting students' transition to safe, competent practice. However, they also note that multiple preceptors, higher preceptor-student ratios than the ideal 1 : 1 ratio [52], workplace and workforce stresses, and inconsistent preparation and support hamper preceptors' ability to facilitate the one-to-one relationship. Furthermore, even though preceptors might desire and be willing to teach and share their knowledge, lack of expertise in teaching and evaluation might cause them stress and create an inability to guide students especially when a student is not progressing well [12, 53].

Although all of these issues are a concern, perhaps a greater challenge is the inability of preceptors to address teaching and learning diversity issues that are related to their role as clinical teachers [54]. For example, differences in ability in cognitive, emotional, developmental, and physical domains affect how learning situations should be approached, planned, and enacted. Differences in age and age cohort can also create educational diversity between a preceptor and student. Indeed, stereotypical portrayals can diminish the unique contributions that an individual brings to the learning encounter. While generalizations cannot be made, belonging to a particular age cohort shapes beliefs, values, abilities, and skill sets that may be unappreciated by individuals from different age cohorts [55]. Last, as the number of international students admitted to nursing programs increase, preceptors and students alike are faced with a variety of challenges. Potential challenges are those of adapting to a change in sociocultural context including social integration and differences in teaching and learning approaches. Another significant issue is that of language diversity. International students may experience difficulty in clinical settings related to the use of terminology, understanding patient requests, providing explanations [56] as well as being able to engage in advocacy behaviours that are perceived as required in complex health environments. Preceptors may be ill equipped to work with these students and as a result may be unable to employ strategies that create a responsive and supportive learning environment [54].

### 4.5. Preceptors

Little research has been conducted on the experience of preceptoring for those registered nurses who volunteer to preceptor as opposed to those who are assigned to precept a nursing student. Of the studies conducted in this area, preceptors experienced role ambiguity accompanied by stress associated with workload issues, and self-efficacy concerns as role models and teachers in clinical interactions with nursing students [57–59].

It could be construed then that although the importance and need for the development of a strong student/preceptor relationship are necessary, current stressors within the preceptorship model inhibit this from taking place. Indeed, adequate time to mentor students in the clinical setting is apparently a key challenge for preceptors [60] and detrimental to their efficacy as role models for students and their subsequent learning [61]. Providing feedback as an evaluative strategy requires knowledge about the feedback loop; however, preceptors often receive little to no specific information on the evaluator role [59]. The ambiguity in their function as preceptors coupled with the lack of recognition by both education and service industries would seem to negate the importance of the role. This may explain the reluctance of many to step forward to take on this role.

### 4.6. Nursing Student

 It should not be surprising that preceptorship is a stressful experience for students [62] since within the complex and fast paced environment of today's clinical practice setting, two strangers, a student and preceptor, are brought together to provide the student with the opportunity to transition from the student to graduate role. Most often the student and preceptor do not choose who they will establish the preceptor relationship with: the pairing is assigned. Students therefore might perceive they have little or no control over the experience. While Yonge et al. [62] encourage a careful assessment of students' readiness for preceptorship, Yonge [63] also acknowledges that some students might not be psychologically prepared for preceptorship. For some students needing to navigate the complex relationships that accompany being socialized into the profession especially when that experience is at a long distance from supervising faculty may be overwhelming.

 Consequently, student preparation for the preceptorship experience is essential so that their learning might be optimized. While students need to take responsibility and accountability for their own learning [1, 64], preparatory course content must also be meaningful and meet student learning needs [65]. Further, it is important that students understand the purpose of the preceptorship experience, how to make the best use of their clinical time [66, 67], and that they develop social competency skills [68]. Social competency involves establishing, maintaining, and developing constructive social relationships with people not only in their personal lives but as well with people in the work setting.

## 5. Conclusion

A critical component in nursing education is clinical practice for student nurses. Although the concept of evidence-based nursing education has found solid footing within the nursing profession and is pervasive in nursing education literature [69], this critique leaves us to question whether it is actually being applied to the preceptorship model in clinical nursing education. The challenges to the preceptorship model described in this paper demonstrate that it is imperative that nurse educators, nursing programs, and leaders in the practice environment engage in critical reflection of the current models of clinical practice education so that programs are able to graduate safe and competent novice registered nurses.
